# Answer changing in multiple choice assessment change that answer when in doubt – and spread the word!

**DOI:** 10.1186/1472-6920-7-28

**Published:** 2007-08-24

**Authors:** Daniel Bauer, Veronika Kopp, Martin R Fischer

**Affiliations:** 1 Schwerpunkt Medizindidaktik, Medizinische Klinik Innenstadt, Klinikum der Universität München, Ziemssenstraße 1, 80336 Munich, Germany

## Abstract

**Background:**

Several studies during the last decades have shown that answer changing in multiple choice examinations is generally beneficial for examinees. In spite of this the common misbelief still prevails that answer changing in multiple choice examinations results in an increased number of wrong answers rather than an improved score. One suggested consequence of newer studies is that examinees should be informed about this misbelief in the hope that this prejudice might be eradicated. This study aims to confirm data from previous studies about the benefits of answer changing as well as pursue the question of whether students informed about the said advantageous effects of answer changing would indeed follow this advice and change significantly more answers. Furthermore a look is cast on how the overall examination performance and mean point increase of these students is affected.

**Methods:**

The answer sheets to the end of term exams of 79 3^rd ^year medical students at the University of Munich were analysed to confirm the benefits of answer changing. Students taking the test were randomized into two groups. Prior to taking the test 41 students were informed about the benefits of changing answers after careful reconsideration while 38 students did not receive such information. Both groups were instructed to mark all answer changes made during the test.

**Results:**

Answer changes were predominantly from wrong to right in full accordance with existing literature resources. It was shown that students who had been informed about the benefits of answer changing when in doubt changed answers significantly more often than students who had not been informed. Though students instructed on the benefits of changing answers scored higher in their exams than those not instructed, the difference in point increase was not significant.

**Conclusion:**

Students should be informed about the benefits of changing initial answers to multiple choice questions once when in reasonable doubt about these answers. Furthermore, reconsidering answers should be encouraged as students will heed the advice and change more answers than students not so instructed.

## Background

Students often believe that the initial answer to a multiple choice question (MCQ) which comes to their mind is the best and that changing an answer, even when another answer option seems better upon reflection, does not lead to a better test score but that it was detrimental to test performance.

However several studies in the past have shown this to be a common misbelief across different levels in various educational domains [1-3].

In 2005 this belief popular among students was also shown to be unwarranted in the area of high stakes medical examinations [4]: Out of the entire set of answers changed, 55% were wrong to right changes (WR), 25% were right to wrong (RW) and 20% were wrong to wrong (WW) with an average net point increase of 1.1% for first changes, consistent with previous research.

As a consequence several studies dealing with answer changing suggest that students should be encouraged to explore their doubts and alter their answers, as they could thereby increase their test scores, provided they do not alter their answer more than once [2,4,5].

However regarding the question whether students would or would not follow this advice, Foote et al. and Prinsell et al. reported no relevant difference in the number of answer changes or gains from answer changing before and after such instruction [6,7]. However in 1982 Sutton reported not only significantly increased numbers of changes but a net gain after systematic instruction on answer changing [8].

To pursue this controversial question this study was conducted to examine whether the advice to change answers after scrutiny was advantageous and if so, whether students in an undergraduate medical education environment would heed the advice and change considerably more answers, thereby improving their test performance as opposed to uninstructed students' changing behaviour in MC-examinations.

## Methods

### Study participants

Seventy-nine 3^rd ^year medical students (45 female and 34 male) from the University of Munich took part in this study which was conducted during the summer of 2004.

### Study design

To confirm the data that changing answers *once *is statistically beneficial, all students were instructed to visibly mark any changes in their choice of answer to a test item as well as the order of said changes.

To answer the question whether students heed the advice to change answers after reconsidering, the students were randomized into two groups. One group was informed that they ought to change answers they had given with the explanation that when in doubt, changing an answer once is statistically beneficial. The other group received no such information. The first group, hereafter referred to as "group with instruction (GI)", consisted of 41 students; the second one, the "group without instruction (GNI)", consisted of 38 students.

Ethical approval for this study was not required by the responsible ethics committee.

### Instruments

#### Test

The students had to answer 78 MCQs from internal (38), general (20) and occupational medicine (20). Each correctly answered question was worth one point, thus a maximum score of 78 points could be achieved.

The question format was single best answer multiple choice: 64 items were one-out-of-five-options questions (A-type), one was a one-out-of-four-options question, one was a one-out-of-six-options question, one was a one-out-of-seven-options questions, two were one-out-of-nine-options questions, three were one-out-of-eleven-options questions and six were one-out-of-fifteen-options questions. On average, the students had 90 seconds for answering each question. The overall test carried a reliability of .85 (Cronbach's Alpha). The mean guessing probability was calculated at 16%.

#### Answer Changes

All students were required to mark their answers and answer changes visibly on the original questionnaire form (e.g. A->C, or C E). The answer to an item ultimately thought to be correct was to be marked on a computer-readable answer sheet. Answer changes were considered if the sequence of answers was either clearly marked on the questionnaire or if the last answer annotated on the questionnaire form differed from the final answer marked on the answer sheet. Unclear changes (39 items or 10.8% of changed items) were omitted from analysis.

### Analysis

The data from the test files was extracted and analysed. Standard descriptive statistical analysis with SPSS 12.0 was applied. Cronbach's Alpha was calculated to check for inner consistency.

## Results

### Value of answer changes

The aggregate random sample was 6162 (79 participants and 78 questions per exam). In total, 323 items were changed once (5.2%).

Altogether 72 of 79 students changed the answer to at least one item. Of these each student on average made 48.2% WR changes (*SD *= 30.3), 21.6% (*SD *= 22.3) RW changes and 30.2% WW choices (*SD *= 27.6).

Each of these 72 students enjoyed an average 1.4 (*SD *= 2.2) point increase from their previous (pre-change) test scores (minimum -2; maximum 8 points) or a percentage increase of 2.5% (*SD *= 4.2) (Table 1). The success rate was calculated as the total number of WR changes minus the number of RW changes.

**Table 1:** Number and effects of first and second/additional answer changes

**Answer change**	**n of students**	**M (SD) of changes (%)**	**M (SD) of changes WR (%)**	**M (SD) of changes RW (%)**	**M (SD) of changes WW (%)**	**M (SD) of point increase**
**First**	72	4.5 (3.0)	2.3 (2.0)	0.9 (1.0)	1.3 (1.3)	1.4 (2.2)
		5.2%	48.2% (30.3)	21.6% (22.3)	30.2% (27.6)	2.5% (4.2)
**Second and additional**	19	1.4 (0.96)	0.21 (0.42)	0.11 (.32)	1.1 (0.94)	0.11 (0.46)
		0.44%	18.4% (38.0)	3.7% (12.1)	77.9% (41.6)	0.19% (0.99)

M, mean; SD, standard deviation.

As a group, students on average achieved 52.4 points (*SD *= 8.8) with a range from 16 to 68 points (theoretical maximum of 78 points).

Second and additional answer changes given by 20 students in a total of 27 items brought a mean point increase of 0.11 (*SD *= 0.46; minimum -1, maximum +1) with 18.4% WR changes, 3.7% RW changes and 77.9% WW answer changes.

### Differences in change rate

To answer the question whether students changed answers more frequently after having been given the advice to do so, the respective changing behaviour of the two groups needs to be analysed. The GI on average changed 4.8 MCQ items once (*SD *= 3.6), whereas the GNI on average changed only 3.3 answers (*SD *= 2.5) (Table 2). This difference in changing behaviour following advice is statistically significant (*t*(77) = 2.24; *p *< .05). The effect size is of middle practical relevance (*d *= .50).

**Table 2:** Answer changes and point increase in GI vs. GNI

	**GI**	**GNI**	
**M (SD) of changes**	4.8 (3.6)	3.3 (2.5)	*p *< .05
**M (SD) point increase**	1.8 (2.5)	0.91 (1.9)	*n.s*.
**% point increase**	2,3%	1,2%	

M, mean; SD, standard deviation

### Differences in performance

The students with instruction gained 1.8 points (*SD *= 2.5) through their increased changing behaviour, whereas the GNI only achieved a point increase of 0.91 (*SD *= 1.9) (Table 2). This difference only just missed the statistical significance (*t*(70) = 1.69; *p *< .10). This result is of middle practical relevance (*d *= 0.40).

The GI got a mean total score of 53.1 points (*SD *= 8.59) on average; the mean total score of the GNI was 51.8 points (*SD *= 9.0). The difference between the two groups concerning their performance was statistically not significant (*t*(77) = 0.51; *n.s*.), the difference in change rate of GI students and GNI students was statistically significant. The effect size is marginal (*d *= 0.15).

## Discussion

This study confirms the data of previous studies which found that students could improve their overall test score by changing initial answers once when in doubt about the answer. It seems that these changes are based on careful consideration, rereading and rethinking the questions and answer options [9].

A look at the mean guessing probability supports this assumption: The probability of a student changing a first answer WR is 25% in a one-out-of-five single best choice (A-type) problem and had a mean of only 20% in this study utilising mixed single best choice items. Contrary to the relatively low guessing probability however is the improvement rate of 48.2%, which in accordance with Lord can be attributed to a recruitment of partial knowledge [10]. Second and additional changes do not contribute to a higher score, suggesting that for second and further changes guessing is the predominant factor.

Results showed that students who had been informed about the benefits of answer changing indeed changed significantly more answers than students who had not been informed (4.8 vs. 3.3 items, *p *< .05), in contrast to data collected by Foote et al and Prinsell et al. [6,7]. Although Foote reported a change in students' attitude towards changing answers to MCQs, no significant change was seen in number of changes or scores before and after intervention through advice on advisability of answer changing. Unlike in Sutton's case, in this study no significant difference in overall performance between the two groups could be determined.

Although students with information performed slightly better overall, the difference was not significant. As students with information had an increased changing behaviour, the point increase of both groups was compared. The difference falls just under the significant level. However, the difference between the groups' point increase adds up to 1.1%. Thus, students could increase their performance by 1.1% if given the advice to change. Considering that the pass grade in medical MCQ-tests in Germany is usually 60%, this is considerable. A 1.1% point increase might not seem a lot at first glance but with increasing item size the effect becomes noticeable. In case of the second German NBE for instance, which consists of 320 items [11], a point increase of 1.1% extrapolates into a 3.5 point increase for students informed about the beneficial effects of answer changing versus those not informed.

McMorris et al. [9] found that "70% of the students indicated they left blanks when initially responding to the items, and thus the extent of answer changing as more generally conceived may have been underestimated in this and previous studies" (p. 169). Combined with the possibility that some students did not mark every change as instructed, since they were taking an exam under stress as well as the relatively low mean guessing probability, it is possible that the overall changing rate was underestimated in this study which suggests that even more answers had been changed than found in this study.

Although this study was based on data from a paper and pencil test, the observed change rate of 5.3% slightly exceeds that of a computer-based study in a similar medical environment by Ferguson et al. [12], for which the overall change rate can be calculated at 4.6%. Both fall within the range of 2% to 9% of answers changed, as reported by Benjamin et al. [1]. The number of WR changes (48.2%) in this study is in full concordance with other studies [1,13].

All along one has to bear in mind though that the simple fact of students knowing answer changing was of some importance might have changed their answer changing behaviour to some extent, as they all had been specifically instructed to mark down answer changes.

Follow-ups, using computer-based exams and including more students and items, could annotate primarily blanked out answers and provide complete documentation of answer changes.

## Conclusion

Both students and teachers should be informed about the statistical benefits of changing first answers after reconsidering when in doubt.

Results also showed that students who had been informed prior to taking the test changed significantly more answers than students who had not been informed. The advice to scrutinize and change answers, when in doubt, is recommended as students heed the advice.

## Competing interests

The author(s) declare that they have no competing interests.

## Authors' contributions

MRF conceived and designed the study. DB participated in the design of the study and acquired the data. VK performed the statistical analysis. VK and DB interpreted the data collected and drafted the manuscript together. All authors read and approved the final manuscript.

## Pre-publication history

The pre-publication history for this paper can be accessed here:



## References

[B1] Benjamin LT, Cavell TA, Shallenberger WR (1984). Staying with Initial Answers on Objective Tests – Is it a Myth?. Teaching of Psychology.

[B2] McMorris RF, Weidemann AH (1986). Answer Changing After Instruction on Answer Changing. Measurement and Evaluation in Counseling and Development.

[B3] Schwarz SP, McMorris RF, DeMers LP (1991). Reasons for Changing Answers: An Evaluation Using Personal Interviews. Journal of Educational Measurement.

[B4] Fischer MR, Herrmann S, Kopp V (2005). Answering multiple-choice questions in high-stakes medical examinations. Medical Education.

[B5] Geiger MA (1991). Changing Multiple-Choice Answers: Do Students Accurately Perceive Their Performance?. Journal of Experimental Education.

[B6] Foote R, Belinky C (1972). It Pays to Switch? Consequences of changing answers on multiple-choice examinations. Psychological Reports.

[B7] Prinsell CP, Ramsey PH, Ramsey PP (1994). Score Gains, Attitudes, and Behaviour Changes Due to Answer-Changing Instruction. Journal of Educational Measurement.

[B8] Sutton JP (1982). Influencing answer changing behaviour: Changing beliefs about the consequences of changing through consultation. Doctoral dissertation.

[B9] McMorris RF, DeMers LP, Schwarz SP (1987). Attitudes, behaviours and reasons for changing responses following answer changing instruction. Journal of Educational Measurement.

[B10] Lord FM (1963). Formula scoring and validity. Educational and Psychological Measurement.

[B11] Bundesministerium für Gesundheit (2002). Approbationsordnung für Ärzte. Bundesgesetzblatt, Jahrgang.

[B12] Ferguson KJ, Kreiter CD, Peterson MW, Rowat JA, Elliott ST (2002). Is That Your Final Answer? Relationship of Changed Answers to Overall Performance on a Computer-based Medical School Course Examination. Teaching and Learning in Medicine, Winter.

[B13] Friedman-Erickson S To Change or Not To Change: The Multiple Choice Dilemma. Proceedings of the Annual Institute of the American Psychological Society on the Teaching of Psychology, June 1994, Washington DC.

